# Clinical and Genetic Advances in Paget’s Disease of Bone: a Review

**DOI:** 10.1007/s12018-016-9226-0

**Published:** 2016-12-19

**Authors:** N. Alonso, I. Calero-Paniagua, J. del Pino-Montes

**Affiliations:** 1 Rheumatology and Bone Disease Unit, Centre for Genomic and Experimental Medicine, Institute of Genetics and Molecular Medicine, University of Edinburgh, Edinburgh, EH4, 2XU UK; 2 Servicio de Medicina Interna, Hospital General Virgen de la Luz, Cuenca, Spain; 3 Unidad de Medicina Molecular, Departamento de Medicina, Universidad de Salamanca, Salamanca, Spain; 4 Servicio de Reumatología. Hospital Universitario de Salamanca, IBSAL, Salamanca, Spain

**Keywords:** Paget’s disease of bone, *SQSTM1* mutations, GWAS, Susceptibility genes, ZiPP study

## Abstract

Paget’s disease of bone (PDB) is the second most common metabolic bone disorder, after osteoporosis. It is characterised by focal areas of increased and disorganised bone turnover, coupled with increased bone formation. This disease usually appears in the late stages of life, being slightly more frequent in men than in women. It has been reported worldwide, but primarily affects individuals of British descent. Majority of PDB patients are asymptomatic, but clinical manifestations include pain, bone deformity and complications, like pathological fractures and deafness. The causes of the disease are poorly understood and it is considered as a complex trait, combining genetic predisposition with environmental factors. Linkage analysis identified *SQSTM1*, at chromosome 5q35, as directly related to the disease. A number of mutations in this gene have been reported, pP392L being the most common variant among different populations. Most of these variants affect the ubiquitin-associated (UBA) domain of the protein, which is involved in autophagy processes. Genome-wide association studies enlarged the number of loci associated with PDB, and further fine-mapping studies, combined with functional analysis, identified *OPTN* and *RIN3* as causal genes for Paget’s disease. A combination of risk alleles identified by genome-wide association studies led to the development of a score to predict disease severity, which could improve the management of the disease. Further studies need to be conducted to elucidate other important aspects of the trait, such as its focal nature and the epidemiological changes found in some populations. In this review, we summarize the clinical characteristics of the disease and the latest genetic advances to identify susceptibility genes. We also list current available treatments and prospective options.

## Introduction

Paget’s disease of bone (PDB) is a chronic disorder characterized by focal or multifocal remodelling and disorganized bone structure [1]. It was firstly described as “osteitis deformans” by Sir James Paget in 1876, prior to the discovery of the X-ray [2]. Nowadays, it is considered a common skeletal condition, representing the most frequent metabolic bone disorder after osteoporosis.

## Epidemiology

PDB appears usually after the age of 40, being slightly more common in men than in women [1, 3]. It has been described almost worldwide, with an irregular geographical distribution [4]. It primarily affects patients of British descent, being common (around 4%) in England [1], areas of Australia, New Zealand [5] and North America [6] and rare (less than 1‰) in Asia, Scandinavia and Africa [7].

Marked differences in prevalence have been found not only among countries but also between areas within the same country [8]. Some regions show a high prevalence of the disease, like the Lancashire focus in UK, with a prevalence of 7% in the population over 55 years [9] and the Vitigudino-Salamanca region in Spain, with a 5.7% prevalence [10].

Several studies suggest that the prevalence and severity of PDB are declining in most but not all of the studied countries [4, 8, 11, 12]. Although the cause of this reduction is not completely understood, environmental changes, such as different migratory patterns, improved diet, sedentary lifestyle and decrease in the exposure to viral infections and zoonoses, might play a role [4].

## Clinical Manifestations

PDB may have a long asymptomatic phase and up to 70% of patients do not present any symptom throughout disease evolution [13].

Clinical manifestations of PDB are pain, bone deformity and features caused by complications, including pathological fractures and deafness [14]. Pain is probably the most common symptom and can be differentiated into primary or secondary. Primary pain is described as dull, deep and predominantly nocturnal. Pain secondary to complications is more frequent than primary pain, especially due to the neurological entrapment or joint deformities [15].

PDB is most commonly located in the pelvis (58–80%), spine (40%), femur (32%) and tibia (16–20%) [16]. In some cases, disease limits to a single bone (monostotic disease), although it often affects several noncontiguous bones (polyostotic disease) [17]. Long bones might bend as a result of the increased bone volume and malleability. Some patients also show skull enlargement and facial deformities, which can transform their physical appearance [14].

The most common complications of PDB comprise arthropathy secondary to an alteration of the subchondral bone, fractures, neurologic compression secondary to bone growth, neurologic dysfunction possibly secondary to vascular steal syndrome, bone hypervascularisation, which may be accompanied by an increased focal heat in the superficial bones as the tibia [18], high output congestive heart failure, hypercalcemia and hypercalciuria in immobilized patients and the tumour transformation of the pagetic bone, commonly into osteosarcoma [19, 20].

## Pathology

PDB manifests with a marked increase in bone turnover, which leads to a larger bone volume [21]. Osteoclasts are mainly affected, experimenting an increase in number and size and containing more nuclei than normal osteoclasts. This results in an elevated metabolic osteolytic activity, coupled with increased bone formation by osteoblasts, which are apparently normal [22].

The pagetic bone lesion could be identified in radiographs as a lytic lesion at the first stage (osteolytic pagetic phase). Then, lesions evolve into a mixture of sclerosis, due to new bone formation by osteoblasts, and osteolysis (mixed phase). In the final stages, sclerotic bone is observed, due to a reduction in bone turnover and cells [23]. Different radiographic patterns could be observed in each stage (Table 1) [24].

**Table 1:** Radiographic changes appearing in each phase of the pagetic lesion

Phase	Radiographic findings
Osteolytic	Osteoporosis circumscripta in skull
	Blade of grass or candle flame signs in long bones
Mixed	Coarsened trabeculae and bony enlargement mixed with osteolytic zones
	Cotton wool appearance of the skull
	Diploic space widening (inner and outer calvaria tables)
	Vertebral frame sign
	Squaring of vertebrae
	Coarse vertebral trabecular thickening
	Ivory vertebrae
	Enlargement of the pubic rami and ischium
Sclerotic	Frontal bone enlargement
	Cortical thickening and sclerosis of the iliopectineal and ischiopubic lines
	Acetabular protrusio
	Lateral curvature of the femur
	Anterior curvature of the tibia
	Looser zones
	Banana and chalk transverse fracture in long bones

Bone turnover is greatly accelerated in the pagetic bone. Therefore, new collagen fibres are placed in a chaotic fashion, unlike the laminar distribution of the adult mature bone. This results in the characteristic mosaic pattern of the pagetic bone, combining an abnormal woven bone, with some areas of lamellar bone, and numerous disorganized cement lines from previous osteolytic phases [25]. In the matrix, the osteoid volume is increased in thickness, but usually without mineralization alterations. The increase in bone turnover leads to an increased number of trabeculae, as it has been described in biopsies from the iliac crest. Numerous connective tissue fibres and hypervascularity are observed in the bone marrow. All the above-mentioned changes induce mechanical tissue modifications that facilitate the bowing deformities and cracks; however, a lower mineralization rate and loss of aligned haversian structures may partly compensate these changes, maintaining resistance to crack growth [26].

## Diagnosis

A diagnosis of PDB is incidental in most cases, when an elevated level of alkaline phosphatase is detected in the absence of liver disease in analyses that were performed for various reasons or the presence of suggestive radiographic changes ordered by other medical problems [16].

Elevation of bone turnover markers reflects changes in bone metabolism. Due to its wide availability, low variability and price, total alkaline phosphatase (ALP) is the most extended marker for PDB activity. A recent meta-analysis suggests that this activity is better monitored by following procollagen type 1 amino-terminal propeptide (P1NP) levels, considering ALP, bone-specific alkaline phosphatase (bone ALP), and C-terminal telopeptide (CTX) as good alternative markers for disease activity in untreated patients, or when P1NP is not available [27].

Plain radiography is often the basis for diagnosis as its features are easily recognizable. The injury does not usually affect the entire bone and the border between healthy and disease areas appears as a lytic image (blade of grass, or candle flame sign in the shaft of long bones (Table 1)). Computed tomography, magnetic resonance and positron emission tomography images may be useful to detect suspected sarcomatous degeneration [28].

Tc-99 bone scan provides images of increased uptake in areas of increased vascularity and osteoblastic activity. Although it is unspecific, it has a high sensitivity to detect lesions, even at the very early stages of PDB that are still not visible on X-ray [29].

Bone biopsy is rarely required for diagnosis, but it may be useful for tumour differential diagnosis. The most characteristic findings are the presence of abnormal trabeculae, irregular cementation lines with a typical “mosaic” image, increase in vascularity and increased number and size of osteoclasts [21].

## Aetiology

The causes of PDB are not well understood and controversies arise with regard to its aetiology [30]. It is considered a complex, multifactorial disease, as a result of a synergistic action between environmental and genetic factors. At an early age, osteoclast precursors could be sensitized by an unknown environmental factor. The genetic conditioning would explain individual susceptibility to finally develop the disease years later [30, 31].

### Genetic Predisposition

PDB shows a strong genetic compound. It has been identified in families since 1883 [32] and it is shown that up to 40% of individuals with PDB have affected relatives [33–36]. It is an autosomal dominant condition [36–38] which appears more commonly in first-degree relatives of affected patients [35].

The first genetic approach to identify the causal gene for PDB was performed in a French-Canadian cohort including 11 families with PDB. Linkage analysis identified the 5q35 locus as associated with the disease (LOD score 3.0) [38]. Subsequent studies isolated sequestosome 1 (*SQSTM1*) as the candidate gene for this locus [39].

In total, seven loci have been associated by linkage analysis with the appearance of classical PDB: 6p21.3 (PDB1 locus) [40, 41], 18q21-22 (PDB2) [42–45], 5q35 (PDB3) [37–39], 5q31 (PDB4) [38], 2q36 (PDB5) [37], 10p13 (PDB6) [37] and 18q23 (PDB7) [46]. The former locus was also associated to rare bone dysplasia familial expansile osteolysis, a rare condition that shares some features with PDB [37].

#### *SQSTM1* Mutations

*SQSTM1* gene maps to chromosome 5q35 and contains eight exons. It encodes p62 protein, a 62-kDa scaffolding protein with three functional regions: an N-terminal region which interacts with kinases, a hinge region and a C-terminal area containing the ubiquitin-binding domain (UBA domain). It targets proteins for degradation through the proteasome pathway [47] and mediates the formation of autophagosome by interacting with LC3 protein [48, 49]. SQSTM1 is also important for bone metabolism, since it is involved in the transduction of the NF-κB pathway, which is key in osteoclast differentiation and function [50].

Germline mutations in *SQSTM1* have been found in ~40% of the PDB familial cases and in 10% of sporadic cases [39, 51]. p.P392L variant was the first *SQTM1* mutation associated with PDB. It has been shown that p.P392 mutation is sufficient to cause PDB in mice, by altering autophagy in osteoclasts [52]. It was initially identified in 46% of familial cases and in 16% of sporadic patients of French-Canadian ascendency [39]. Similarly, it was identified in 19% of familial and 8.9% of sporadic cases in British patients [51], as well as in the Belgian [53], Italian [54] and American populations [55] (Table 2). This mutation was also commonly detected in the Chinese population, where the appearance of Paget’s disease is rare. Reported cases in this population showed similar demographic and clinical features than in Caucasian patients [56, 57]. The above findings suggest that p.P392L is a mutation hotspot. Several other hotspots have been identified in the protein, mainly in the UBA domain [58–61].

**Table 2:** *SQSTM1* mutations identified in patients with classical PDB

Gene	Mutation	Protein change	Domain affected	Population	Ref
SQSTM1	T1046A	D335E	–	Italian	Falchetti et al., 2009 [59]
	T1085A	S349 T	KIR	American (German descent)	Michou et al., 2011 [60]
	C1090T	P364S	P2	Australian	Rea et al., 2009 [61]
	A1132T	K378X	–	Australian	Rea et al., 2006 [132]
	C1182T	A381V	–	Italian	Falchetti et al., 2009 [59]
	C1190A	Y383X	–	Italian	Gennari et al., 2010 [133]
	C1200T	P387L	UBA	USA (mixed European descent), Italian	Johnson-Pais et al., 2003 [55], Longato et al., 2014 [134]
	G1205C	E389Q	UBA	American	Beyens et al., 2006 [104]
	C1209T	A390V	UBA	Italian American	Michou et al., 2011 [60]
	IVS7+1G>A	A390X	UBA	French	Collet et al., 2007 [58]
	C1215T	P392L	UBA	French-Canadian, Italian, New Zealand, USA (mixed European descent), British, Netherlands, Australian, Chinese, Polish-American, Irish-Italian, African-American	Laurin et al., 2002 [39]; Falchetti et al., 2004 [54]; Cundy et al., 2011 [135]; Johnson-Pais et al., 2003 [55]; Hocking et al., 2002 [51]; Eekhoff et al., 2004 [35]; Good et al., 2004 [136]; Gu et al. 2012 [56]; Michou et al., 2011 [60]
	1210delT	L394X	UBA	USA (mixed European descent)	Johnson-Pais et al., 2003 [55]
	1225insT	E396X	UBA	British, Australian, New Zealand	Hocking et al., 2002 [51]; Rea et al., 2006 [132]; Cundy et al., 2015 [117]
	T1229G	S397A	UBA	Italian	Falchetti et al., 2009 [59]
	T1235C	S399P	UBA	Netherlands	Eekhoff et al., 2004 [35]
	C1238T	Q400X	UBA	British	Visconti et al., 2010 [63]
	A1241G	M401V	UBA	Italian	Gennari et al., 2010 [133]
	A1250G	M404V	UBA	Italian, British	Falchetti et al., 2004 [54]; Hocking et al., 2004 [62]
	T1251C	M404T	UBA	Netherlands	Eekhoff et al., 2004 [35]
	G1271A	G411S	UBA	British	Hocking et al., 2004 [62]
	C1277T	L413F	UBA	French	Collet et al., 2007 [58]
	T1290A	L417Q	UBA	American (Russian Jewish ancestry)	Michou et al., 2011 [60]
	1307insT	D423X	UBA	Italian	Falchetti et al., 2009 [59]
	T1311G	I424S	UBA	British	Visconti et al., 2010 [63]
	G1312A	G425E	UBA	Italian, Netherlands	Gennari et al., 2010 [133]; Eekhoff et al., 2004 [35]
	G1313A	G425R	UBA	Italian	Falchetti et al., 2004 [54]
	unknown	A426V	UBA	unknown	Rea et al., 2013 [137]^a^
	C1320A	A427D	UBA	Italian, British	Gennari et al., 2010 [133]; Goode et al., 2014 [138]
1p13.3 (CSF1)	rs10494112	Intergenic	–	British, Australian, New Zealand, Italian, Spanish	Albagha et al., 2010 [74]
	rs499345	Intergenic	–	British, Australian, New Zealand, Italian, Spanish	Albagha et al., 2010 [74]
	rs484959	Intergenic	–	British, Australian, New Zealand, Italian, Spanish	Albagha et al., 2010 [74]
18q21.33 (TNSFRF11A)	rs2957128	Intergenic	–	British, Australian, New Zealand, Italian, Spanish	Albagha et al., 2010 [74]
	rs3018362	Intergenic	–	British, Australian, New Zealand, Italian, Spanish	Albagha et al., 2010 [74]
OPTN	rs1561570	Intronic	–	British, Australian, New Zealand, Italian, Spanish	Albagha et al., 2010 [74], Obaid et al., 2015 [91]
7q33 (NUP205)	rs4294134	Intronic	–	British, Australian, New Zealand, Italian, Spanish, Belgian, Dutch	Albagha et al., 2011 [73]
15q24.1 (PML)	rs5742915	p.F645L	–	British, Australian, New Zealand, Italian, Spanish, Belgian, Dutch	Albagha et al., 2011 [73]
8q22.3 (DC-STAMP)	rs2458413	Intronic	–	British, Australian, New Zealand, Italian, Spanish, Belgian, Dutch	Albagha et al., 2011 [73]
TM7SF4	C1189T	L397F	–	French-Canadian	Beauregard et al., 2014
CTHRC1	372+259A>G	Intronic	–	French-Canadian	Beauregard et al., 2014
RIN3	1-926A>G	Promoter	–	British	Vallet et al., 2015 [31]
	-21C>A	5’UTR	–	British	Vallet et al., 2015 [31]
	C422T	A141V	SH2	British	Vallet et al., 2015 [31]
	C691T	R231C	–	British	Vallet et al., 2015 [31]
	C751A	Q251K	Pro-rich	British	Vallet et al., 2015 [31]
	C835T	R279C	Pro-rich	British	Vallet et al., 2015 [31]
	T866C	L289P	Pro-rich	British	Vallet et al., 2015 [31]
	T874C	C292R	Pro-rich	British	Vallet et al., 2015 [31]
	C880T	P294S	Pro-rich	British	Vallet et al., 2015 [31]
	G916C	A306T	Pro-rich	British	Vallet et al., 2015 [31]
	C1156T	P386S	Pro-rich	British	Vallet et al., 2015 [31]
	G1280A	R427Q	Pro-rich	British	Vallet et al., 2015 [31]
	C1429T	P477S	Pro-rich	British	Vallet et al., 2015 [31]
	G1838C	G613A	VPS9	British	Vallet et al., 2015 [31]
	G2311A	D771N	VPS9	British	Vallet et al., 2015 [31]
	T2377T	Y793H	VPS9	British	Vallet et al., 2015 [31]
ATG16L1	A898G	T300A	–	Spanish	Usategui-Martin et al., 2015 [102]
ATG5	rs2245214	Intronic	–	Spanish	Usategui-Martin et al., 2015 [102]
ATG10	C635T	T212M	–	Spanish	Usategui-Martin et al., 2015 [102]
ZNF687	C2810G	P937R	–	Italian and multiethnic American	Divisato et al., 2016 [115]

^a^Mutation reported in a review. The original research article was not found

To date, 28 different mutations in SQSTM1 have been reported, producing 21 aminoacid substitutions and various truncating mutations affecting the UBA domain of the protein (Table 2). Patients with truncating mutations showed a more severe phenotype than the individuals with missense mutations [61, 62]. Most of the patients present a single mutation in the gene, although several cases have been identified with compound heterozygous mutations [35, 58, 63] and homozygous p.P392L [34].

Only four mutations were identified out of the UBA domain of SQSTM1 (Table 2) [58–61]. These mutations, like p.S349T, also increase NF-κB signalling [61]. This occurs through the reduction in binding SQSTM1 to Keap1, which reduces the activity of Nrf2. Alteration of the Nrf2 function could produce an increase in the oxidative response genes, contributing to the appearance of PDB. Loss of Nrf2 in vivo negatively affects osteoblast differentiation and matrix formation, and it has been proposed that mutations in SQSTM1 could produce alterations in bone remodelling as seen in PDB patients through altering the Nrf2 cellular activity [64].

It is known that PDB is a focal disease showing asymmetric distribution, however, the cause is still unknown. It has been hypothesised that somatic mutations at the early stage of the zygote could be responsible for the mosaicism detected in the patients. Consistently, several studies found p.P392L variant as a somatic mutation in *SQSTM1* in the affected bones from two unrelated patients, but not in peripheral blood [65], or restricted to monocytes [66].

Mutations in *SQSTM1* have also been reported in other diseases, like amyotrophic lateral sclerosis, in cohorts with familiar, sporadic and frontotemporal dementia—ALS, from Europe, the USA and Japan [67–71]. Among the rare or novel coding mutations found, some of them pathogenic, p.Pro392Leu and p.Glu155Lys, were also identified. The patient carrying p.P392L mutation developed Paget’s disease, as well as the father of the proband carrying p.Glu155Lys mutation [71].

#### Genome-Wide Association Studies (GWAS)

*SQSTM1* mutations have been found in only 20–50% of PDB patients, therefore high-throughput screening techniques, like genome-wide association studies, were used to identify unknown candidate genes [72, 73]. An initial study carried out by Albagha et al. analysed 1250 *SQSTM1*-ve cases and 1537 controls and identified six SNPs in chromosomes 1, 10 and 18 associated with the disease (*p* values ranging from 1.86e-11 to 5.38e-24) (Table 2) [74]. Risk allele carriers have ~70% of increase in predisposition to develop the disease [74].

Chromosome 1p13 highlighted a recombination area where only *CSF1* gene was located. This gene encodes M-CSF, the macrophage colony-stimulating factor, involved in osteoclast formation and survival [75, 76]. An increase in serum M-CSF has been detected in patients with PDB [77]. The causal variants in this gene that predispose to PDB remain unknown, but it is suggested that they could induce PDB by increasing osteoclast formation, via CSF1 activity [78].

Individuals carrying the risk allele of SNP rs1561570, located in chromosome 10p13, showed an increase of ~60% in developing the disease [74]. This region has been previously detected by linkage analysis, defined as PDB6 locus [37], but the causal gene was not isolated. GWAS allowed to identify a recombination area where Optineurin (*OPTN*) gene is located. *OPTN* plays a role in glaucoma [79], but no function has been previously reported in bone metabolism.

Chromosome 18q21.33 corresponds to PDB7 locus, previously identified in some families by linkage analysis [46]. Top GWAS SNPs were located in an intergenic region close to *TNFRSF11A* gene. It encodes RANK, a receptor protein for RANKL which activates NF-κB signalling. RANK is a key protein for osteoclast differentiation and function, and its disruption leads to an osteopetrotic phenotype in mice [80]. Recent studies have shown that genetic variability of genes such as *TNFRSF11A/RANK* could increase the severity of the disease in patients carrying a mutation in *SQSTM1* [81]. Other syndromes with similar clinical characteristics as PDB were also associated with mutations in RANK gene, like familial expansile osteolysis, early-onset familial PDB and expansile skeletal hyperphosphatasia [44, 45, 82].

Enlarged GWAS analysis in 2223 *SQSTM1*-ve PDB cases and 4601 controls confirmed the previous GWAS findings and identified four novel signals in chromosomes 7, 8, 14 and 15 (Table 2) [73]. The strongest signal at 7q33 was driven by rs4294134 variant, located in an intronic region of *NUP205* gene. It encodes nucleoporin 205 kDa, a component of the nuclear pore involved in transport processes [83]. However, its role in the bone is still unknown.

The signal on chromosome 8q22.3 appointed to an 18-kb LD block covering the whole transmembrane 7 superfamily member 4 (*TM7SF4*) gene. This gene encodes DC-STAMP protein, involved in the fusion of osteoclasts precursors to form mature osteoclasts [84]. Expression of DC-STAMP is essential for osteoclast formation [85]. Genetic variants predisposing to PDB could enhance the expression of DC-STAMP, to generate the large multinucleated pagetic osteoclasts [73].

SNP on chromosome 14q32.12 also appoints to a novel gene in PDB, *RIN3* [73]. It encodes Ras and Rab interactor 3, involved in vesicular trafficking [86, 87]. Its functionality and association with PDB is discussed in “Novel genes associated with PDB”.

Chromosome 15q24.1 also constitutes a new susceptibility locus for PDB. Rs5742915, a missense change (p.Phe645Leu) of promyelocytic leukaemia gene (*PML*), showed the highest association [73]. This gene is involved in TGF-β signalling and involved in the regulation of bone remodelling [88]. *GOLGA6A* gene, a member of the golgin family, is located in the same area and could not be completely discarded. Its role in bone metabolism is unknown, but mutations in other members of the same family produce a severe form of osteoporosis [89] and lethal skeletal dysplasia [90].

#### Novel Genes Associated with PDB

Genome-wide association studies allowed to identify most of the genetic loci involved in the development of the disease. To date, only two GWAS regions have been studied in detail: chromosome 10p13 (*OPTN* gene) [91] and chromosome 14q32.12 (*RIN3* gene) [31].

Chromosome 10p13 highlights Optineurin gene, involved in NF-κB signalling regulation [92], autophagy and immunity [93]. SNP rs1561570 was the strongest signal in GWAS for this locus (*p* value = 4.37e-38, OR = 1.67 [1.54–1.810]) and was an expression quantitative trait locus (eQTL), reducing the levels of OPTN in T-allele carriers [91]. Mouse knockdown model for *optn* showed that the gene acts as a negative regulator of osteoclast differentiation in vitro [91]. OptnD477N/D477N knockout mice formed more hypernucleated osteoclasts compared to the wild type. Osteoblasts from these mice showed a reduction in their role to promote osteoclast differentiation. Osteoclast results were supported in vivo. An increase in bone resorption in these mice is thought to be coupled with an increase in bone formation, therefore, no bone loss was found. After RANKL stimulation, an increase in NF-κB activation was detected in these mice. The inhibitory effect of Optn on osteoclasts is mediated by a CYLD-dependent pathway, which is important for the inhibition of NF-κB activation. Optn also inhibits osteoclast differentiation by modulating INF-β signalling pathway. Knockdown and Knockout Optn mice showed enhanced osteoclast differentiation. Rs1561570 SNP in Optn gene increases susceptibility to PDB by reducing OPTN expression [91].

Chromosome 14q32.12 was strongly associated to PDB (*p* value = 2.55e-11, OR = 1.44 [1.29–1.60]) in the European population, appointing to Ras and Rab interactor 3 (*RIN3)* as the causal gene, since small GTPases, like Ras and Rab, are important for osteoclast function [94, 95], and molecules involved in vesicular trafficking cause syndromes with PDB-like characteristics, namely inclusion body myopathy with early onset Paget’s disease and frontotemporal dementia [96]. Deep sequencing of the 14q32 locus in 121 PDB patients and 49 controls from the UK identified p.R279C, in strong LD with the GWAS signal rs10498635, as the most probable causal variant for this locus (*p* value = 1.4e-9, OR = 0.64 [0.55–0.74]). Two other common *RIN3* variants (p.H215R and p.T425M) were also detected, but association was inconclusive. In addition, 13 rare missense variants were identified in these patients, affecting either the structured domains (SH2 and VSP9) or the proline-rich domain (Table 2). A combination of these rare variants was associated with an increased risk of presenting the disease [31]. Analysis of mouse tissues showed that RIN3 expresses higher in the lung, followed by bone tissue, with a tenfold expression in osteoclasts compared with that in osteoblasts. These findings suggest that *RIN3* could be involved in the pathogenesis of PDB by affecting the osteoclast function in these patients [31].

A missense variant (L408P) in *CSF1* gene was detected in a 30-year-old patient with juvenile Paget’s disease, a rare PDB-like syndrome appearing in early stages of life [97]. The patient also carried a missense variant D349G in *TMSF4* gene. A rare variant (allele frequency < 0.05) rs62620995 in *TM7SF4* gene was identified in a French-Canadian cohort of PDB patients, together with rs62641691 variant in *CD276* (Table 2) [98]. Rs62620995 (p.Leu397Phe) could increase the activity of DC-STAMP, altering its expression or its internalization [99].

#### Other Factors

SQSTM1 protein directs ubiquitinated molecules to degradation in autophagolysosomes. It interacts with autophagy protein LC3, located in the ruffle border of the osteoclasts [48]. Besides, other autophagy proteins regulate osteoclastic bone resorption (ATG5, ATG7 and ATG4B), although the SQSTM1-mediated autophagy role in osteoclasts remains to be confirmed [100]. Alterations in autophagosomes have been found in other diseases with a pagetic component, such as inclusion body myopathy, PDB and FTD, linked to a mutation in the VCP gene [101]. Analysis of a Spanish cohort of 238 PDB patients showed that polymorphisms in genes associated with autophagosome formation, ATG16L1 and ATG5, were linked to an increased risk of developing PDB, whilst a polymorphism in ATG10 decreased the risk of suffering the condition (Table 2) [102].

Splicing site mutation in *SQSTM1* have been reported [103, 104] and alternative splicing has been involved in the development of bone diseases, such as *TCIRG1*-linked autosomal recessive osteopetrosis [105]. Alternative splicing in six genes (*LGALS8*, *RHOT1*, *CASC4*, *USP4*, *TBC1D25* and *PIDD*), not previously associated with the disease, but associated with TRAF6 ubiquitination [106], apoptosis [107–110] and autophagosome maturation [111], have been associated with PDB.

#### Genetics of PDB Severity

Results from the genome-wide association analysis helped to build up a risk allele score for severity of disease. In patients without *SQSTM1* mutations, a combination of risk GWAS alleles in the highest tertile was associated with a 27% increase in disease extent, defined by the number of affected bones, and 25% increase in disease severity score, which includes complications secondary to the disease. *SQSTM1*+ve patients showed a highly significant increase in disease extent, severity and number of previous treatments received [112].

In a reduced number of cases, neoplastic transformation appears in the pagetic bones, producing osteosarcoma or, even less frequently, giant cell tumours [113, 114]. This is a serious condition since about 80% of patients diagnosed with GCT die in 10-year time. Analysis of a large family with 14 members affected by PDB, *SQSTM1*-ve, and four of them presenting giant cell tumours identified a heterozygous missense mutation in the *ZNF687* gene (p.P937R) in all unrelated PDB patients and replicated in two families with PDB history. This variation was identified as a founder mutation since it originated from a unique haplotype and segregated in all but one GCT/PDB-affected individual in the study. These results confirmed that p.P937R is necessary and sufficient for the development of GCT in PBD patients. Authors also found a small group of familial PDB patients carrying this mutation, associated with a more severe phenotype than PDB patients without the mutation, with a polyostotic disease and earlier onset of disease. ZNF687 encodes a C2H2 zinc finger protein involved in the transcriptional regulator complex Z3. It is widely expressed, including in the bone, where it is upregulated in osteoclast and osteoblast differentiation in zebrafish model. This gene is located downstream of NF-kB. Mutation p.P937R is a gain-of-function change, producing an accumulation of the protein in the nucleus and subsequent transcription of the pathway downstream. Osteoclast derived from patients carrying this mutation presented an increased size and number of nuclei [115].

### Environmental Triggers

Genetic predisposition plays a crucial role in the development of PDB; however, some studies have found that children whose parents carry *SQSTM1* mutations do not always develop the disease, or they present a large delay in the appearance of the symptoms [34, 116, 117]. Similarly, mice expressing pP392L SQSTM1 mutation showed an increased number of osteoclasts and progressive bone loss, but osteoblasts were not increased and, therefore, did not present any visible pagetic lesion [118]. These findings, together with the reported changes in the incidence of PDB, support the role of environmental factors in the development of the disease. A persistent viral infection was proposed after observing intracellular inclusions in osteoclasts, similar to measles nucleocapsids (MVNP) [119, 120]. The nature of these bodies is still controversial, since some groups have not found any connexion [121]. It has been suggested lately that they could be protein aggregates resulting from the dysregulation of the autophagy system [122, 123]. However, recent studies have shown that the MVNP protein is associated with the upregulation of IL-6 and IGF1 in osteoclasts from mouse models and PDB patients, which could suggest a role for measles virus in the alteration of bone formation seen in these patients [124, 125].

PDB has also been linked to other factors such as poor calcium and vitamin D intake, consumption of uncontrolled beef meat during childhood [126], consumption of not purified water [10], contact with dogs during early years [127], an excessive mechanical loading on the skeleton and exposure to some environmental toxics [128].

## Treatment

The main and the only absolute indication for treatment with clear clinical evidence of PDB is pain in the affected bone [29]. In several clinical trials, bisphosphonates were effective in managing pain in PDB patients. Zoledronic acid is the most potent drug and is currently the first choice of treatment [129]. Calcitonin is effective in reducing pain and expression of bone formation and resorption markers, although its power is clearly lower than that of bisphosphonates; thus, it is rarely used nowadays [130]. Denosumab is a potent inhibitor of bone resorption and has been reported to decrease disease activity in one PDB patient [131], but it has not been tested yet in clinical trials for PDB. Orthopaedic surgery is recommended mainly for bone pagetic fractures, spinal stenosis or pagetic osteoarthritis.

Treatment of PDB patients showing only biochemical activity but no pain is under debate. To date, there is not enough evidence on preventive treatment of complications in asymptomatic patients. The PRISM trial and its extension showed no beneficial effect on the quality of life, fractures, orthopaedic surgery or deafness in patients treated repeatedly with bisphosphonates [33]. An international randomized clinical trial led by Prof Stuart Ralston, at the University of Edinburgh, UK (Zoledronate in the prevention of Paget’s: the ZiPP study, ISRCTN11616770) is currently in progress to detect the effect of bisphosphonate treatment in *SQSTM1*+ve individuals who have not developed any symptom.

## Conclusion

Paget’s disease of bone is a common disorder resulting from a combination of genetic and environmental factors. To date, clinical, laboratory or radiographic features have been used to identify the disease and provide treatment, although the guidelines to prescribe zoledronic acid are still under debate.

*SQSTM1* mutations are associated with susceptibility to develop PDB. However, only 40% of familial PDB and 10% of sporadic PDB patients present alterations in this gene. Latest advances in the genetics field identified seven other genes predisposing to the disease. It has been shown that genetic information may constitute a good tool to manage presymptomatic patients. A risk allele score has been developed using the information from all PDB loci, to successfully detect an accumulative risk to develop a more severe disease when carrying a large number of risk alleles. In addition, forthcoming results from the ZiPP study will be crucial to determine prophylactic treatment based on genetic profiling may contribute to prevent skeletal complications associated with PDB.

However, despite the great genetic advances, further research is needed to elucidate other aspects of the disease, including its focal nature, and the changes in severity and prevalence observed in some populations.
